# Effects of excipients on the interactions of self-emulsifying drug delivery systems with human blood plasma and plasma membranes

**DOI:** 10.1007/s13346-024-01541-w

**Published:** 2024-02-27

**Authors:** Bao Le-Vinh, Nguyet-Minh Nguyen Le, Thi Nhu Quynh Phan, Hung Thanh Lam, Andreas Bernkop-Schnürch

**Affiliations:** 1 Department of Pharmaceutical Technology, Institute of Pharmacy, University of Innsbruck, Innrain 80/82, Innsbruck, 6020 Austria; 2 Department of Industrial Pharmacy, Faculty of Pharmacy, University of Medicine and Pharmacy, Ho Chi Minh, 700000 Viet Nam; 3 Department of Pharmaceutical Technology, Faculty of Pharmacy, Can Tho University of Medicine and Pharmacy, Can Tho, Viet Nam

**Keywords:** SEDDS, Protein binding, Plasma membrane disruption, Unsaturated fatty acid, Protein corona, Size exclusion chromatography

## Abstract

Due to its versatility in formulation and manufacturing, self-emulsifying drug delivery systems (SEDDS) can be used to design parenteral formulations. Therefore, it is necessary to understand the effects of excipients on the behavior of SEDDS formulations upon parenteral administration, particularly their interactions with blood plasma and cell membranes. In this study, we prepared three neutrally charged SEDDS formulations composed of medium-chain triglycerides as the oil phase, polyoxyl-35 castor oil (EL35) and polyethylene glycol (15)-hydroxystearate (HS15) as the nonionic surfactants, medium-chain mono- and diglycerides as the co-surfactant, and propylene glycol as the co-solvent. The cationic surfactant, didodecyldimethylammonium bromide (DDA), and the anionic surfactant, sodium deoxycholate (DEO), were added to the neutral SEDDS preconcentrates to obtain cationic and anionic SEDDS, respectively. SEDDS were incubated with human blood plasma and recovered by size exclusion chromatography. Data showed that SEDDS emulsion droplets can bind plasma protein to different extents depending on their surface charge and surfactant used. At pH 7.4, the least protein binding was observed with anionic SEDDS. Positive charges increased protein binding. SEDDS stabilized by HS15 can adsorb more plasma protein and induce more plasma membrane disruption activity than SEDDS stabilized by EL35. These effects were more pronounced with the HS15 + DDA combination. The addition of DDA and DEO to SEDDS increased plasma membrane disruption (PMD) activities, and DDA (1% w/w) was more active than DEO (2% w/w). PMD activities of SEDDS were concentration-dependent and vanished at appropriate dilution ratios.

**Graphical abstract:**

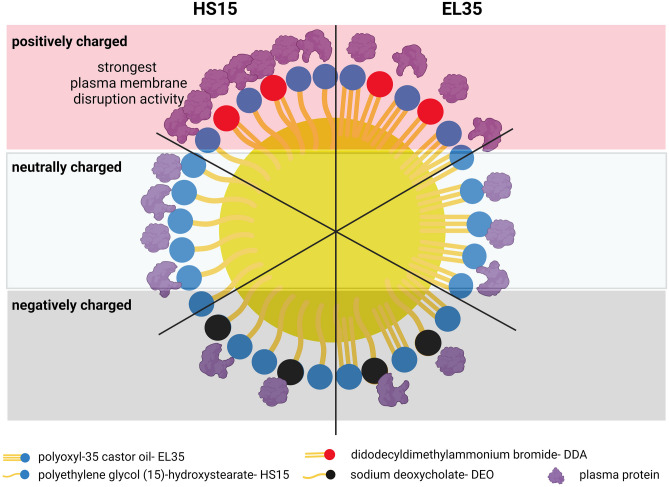

**Supplementary Information:**

The online version contains supplementary material available at 10.1007/s13346-024-01541-w.

## Introduction

Self-emulsifying drug delivery systems (SEDDS) are isotropic mixtures of oil, surfactants, and co-solvents/co-surfactants that have been used for the design of drug formulations to improve the oral absorption of highly lipophilic drug compounds [1] and peptide drugs [2, 3], and reduce food effect [4]. Unlike conventional emulsions, SEDDS has the advantage of being able to form emulsions by mildly mixing the SEDDS preconcentrate and an aqueous medium which could be either gastrointestinal fluids or a diluent before use. Active components, especially compounds that are prone to hydrolysis, are supposed to be more stable in the water-free SEDDS preconcentrate. Moreover, the manufacturing of SEDDS on an industrial level is economical and simpler than other nanocarriers like emulsions, liposomes, or polymer-based nanoparticles since it is almost like the preparation of solution [3]. Currently, most SEDDS formulations available on the market are licensed for oral drug delivery [5]. Recently, SEDDS has been utilized to formulate vaccine adjuvants for intramuscular injection [6–8], and used for intravenous injection [9]. There has been some research on the physicochemical interaction of emulsions and plasma proteins [10, 11], but they mostly focused on perfluorocarbon emulsions as artificial oxygen carriers to replace homologous blood. Moreover, SEDDS and conventional emulsions are formulated in quite different principles regarding the type of excipients, composition, and dispersion force. Therefore, there is a need to understand how SEDDS droplets that are intramuscularly or intravenously administered might interact with blood and cell membranes.

Surface charge, surface chemistry and size of nanoparticles can strongly affect protein binding affinity and specificity [12, 13]. Protein binding onto nanoparticles can cause changes in their size and surface charge, and consequently has a strong impact on their distribution throughout the body, elimination, phagocytosis, cellular uptake at injection site, and intracellular signaling [14, 15]. It has been shown that high surface charge densities on liposomes can result in faster blood clearance and reticuloendothelial system (RES) capture while the neutral charges can contribute to the extended blood circulation and reduced RES clearance [16, 17]. Besides, nonionic and ionic surfactants that play a pivotal role in determining the emulsion surface properties may cause hemolysis [18] and affect the safety profile of SEDDS formulation. The understanding of how SEDDS excipients affect surface properties of SEDDS emulsion droplet can benefit the SEDDS formulation design to achieve desired biological activity.

In this study, we investigated the impact of different surfactants and surface charges on the association of SEDDS and blood plasma proteins, and the interaction of SEDDS and cell membranes. We prepared three SEDDS formulations with different particle sizes using different mass ratios of the nonionic surfactants, polyoxyl-35 castor oil and polyethylene glycol (15)-hydroxystearate. These surfactants are approved for uses in oral, parenteral, and ophthalmic formulations. We added the cationic surfactant, didodecyldimethylammonium bromide (DDA), and the anionic surfactant, sodium deoxycholate (DEO), to SEDDS formulations to make them positively and negatively charged, respectively. SEDDS was incubated with blood plasma and then separated from the mixture by size exclusion chromatography (SEC). Particle size, zeta potential (ZP) and protein binding amount of recovered SEDDS were measured and compared with the control, SEDDS incubated with phosphate buffered saline (PBS). Furthermore, interaction of SEDDS and cell membranes was evaluated using red blood cells as a model. The ability of SEDDS to disrupt erythrocyte membrane was evaluated by released hemoglobin assay.

## Materials and methods

### Materials

Labrafac^®^ Lipophile WL 1349 (MCT), mixture of medium-chain triglycerides of caprylic and capric acids, was kindly provided by Gattefossé. Capmul^®^ MCM EP/NF (MCM), mixture of mono- and di-glycerides of caprylic and capric acids, was kindly provided by Abitec. Kolliphor® HS 15 (polyethylene glycol (15)-hydroxystearate- HS15), Kolliphor^®^ EL (polyoxyl-35 castor oil- EL35), propylene glycol (PG), didodecyldimethylammonium bromide (DDA), sodium deoxycholate (DEO), sterile Dulbecco’s phosphate buffered saline pH 7.4 (PBS), fluorescein isothiocyanate isomer I (FITC) and pyrene were purchased from Sigma Aldrich, Austria. Sephacryl^®^ S400-HR suspension (in 20% aqueous ethanol) with particle size 25–75 μm in wet state and fractionation range of globular proteins 2 × 10^4^ – 8 × 10^6^ was purchased from Sigma Aldrich, Austria. Micro BCA™ protein assay kit was purchased from Thermo Fisher Scientific, Austria. Human whole blood and frozen human plasma were kindly donated by the Blutbank, Tirol Kliniken GmbH, Innsbruck, Tirol, Austria. During whole blood donation, the anticoagulant solution CPD (citrate-phosphate-dextrose) was used to prevent clotting and maintain the shelf life of the blood products.

### SEDDS preconcentrate preparation

SEDDS preconcentrates were composed of MCT as the oil phase, MCM as the co-surfactant, HS15 or EL35 as surfactants, and PG as the co-solvent (Table 1). All ingredients were weighed in a 2-mL Eppendorf tube, vortexed for 15 s and shaken at 37 ^o^C and 750 rpm for 12 h (ThermoMixer C, Eppendorf, Germany). To prepare positively charged SEDDS (Fpos) or negatively charged SEDDS (Fneg), 1% w/w DDA or 2% w/w DEO was added to SEDDS preconcentrates, respectively. To detect SEDDS oil droplets in fractions eluted after SEC, the fluorescent marker pyrene (logK_OW_ 4.88, 𝜆_ex_/𝜆_em_ 339 nm/391 nm) was loaded in SEDDS preconcentrates at 0.1% w/w.

### Size and zeta potential measurement

Particle hydrodynamic size, polydispersity index (PdI) and ZP were measured by dynamic light scattering technique (Malvern Zetasizer Nano ZSP). Size was measured with backscatter (173°) detection mode. For particle size analysis, SEDDS preconcentrates were diluted 1:30 v/v in PBS and gently mixed for 10 s using a vortex mixer. Triplicate measurements were carried out at 25 ^o^C. Samples in PBS were further diluted 5 or 10 times in water for ZP measurement which was performed using a Dip cell (Malvern ZEN 1002).

### Interaction of SEDDS and plasma

Pyrene loaded SEDDS preconcentrate was diluted 1:30 w/v in PBS by gently mixing for 10 s using a vortex mixer to form nanoemulsion. Human plasma was mixed with FITC labeled human plasma at 2:1 ratio to detect the elution of plasma proteins. FITC emits 529 nm light when excited by 495 nm light that would avoid signal interference with pyrene fluorescence. To 50 µL of the nanoemulsion, 100 µL of mixed human plasma was added and the mixture was briefly vortexed and incubated at 37 °C with gently shaking for 15 min (ThermoMixer C, Eppendorf, Germany). Thereafter, the mixture was cooled down to room temperature for 30 min before loading onto SEC column to separate free plasma protein and SEDDS droplets.

For FITC labeled plasma preparation, plasma proteins were reacted with FITC [19]. Briefly, plasma was thawed in the fridge and diluted 1:3 v/v in freshly prepared 0.1 M sodium carbonate buffer pH 9. FITC solution of 1 mg/mL was freshly prepared by dissolving FITC in anhydrous ethanol. To 1 mL of the diluted plasma solution, 50 µL of FITC solution was slowly added in 5 µL aliquots while gently and continuously stirring. After all the FITC solution has been added, the reaction mixture was incubated in the dark for 3 h at 4 °C. Thereafter, reaction mixture was dialyzed (Spectra-Por^®^ Float-A-Lyzer^®^ Molecular weight (MW) cut-off 3.5-5 kDa) against PBS in the dark for 4 h at 4 °C with dialysis buffer changed every hour. Finally, FITC labeled plasma was lyophilized (Christ Gamma 1–16 LSC, Germany) and reconstituted with water to the original plasma volume before use.

### Separation of SEDDS droplet and plasma protein

Gravity-flow SEC using Sephacryl^®^ S-400 HR resin was utilized to separate SEDDS droplets and plasma protein. Sephacryl^®^ S-400 HR was packed in a 10 mL pipette by slurry packing method forming a resin bed of 0.8 cm width and 9 cm length. Fifty µL of the SEDDS-plasma mixture was loaded onto the column and eluted with PBS at a flow rate of 0.05 mL/min. Fractions of 200 µL were collected. The elution profile of SEDDS droplets and plasma protein were monitored by measuring the fluorescence signals of pyrene (𝜆_ex_/𝜆_em_ 339 nm/391 nm) and FITC (𝜆_ex_/𝜆_em_ 495 nm/529 nm), respectively, from each fraction. As controls, SEDDS incubated with PBS was prepared and run through the SEC column.

### Quantitation of amount of total protein associated with recovered SEDDS

Total protein amount in each fraction was determined by the micro-BCA™ Protein Assay Reagent Kit following the manufacturer’s instructions. Aliquot from each fraction was diluted 5 times for the assay. A standard curve was prepared by plotting the absorbance values at 562 nm wavelength against the concentrations of bovine serum albumin standards. The linear range was 0-200 µg/mL. The amount of total protein associated with SEDDS (µg protein/mg SEDDS) was calculated for the fractions containing recovered SEDDS. The amount of SEDDS (mg) in SEDDS containing fraction was determined by a fit logarithm curve obtained by a serial dilution of the corresponding pyrene loaded SEDDS.

### Hemolysis test to evaluate interaction with cell membrane

In vitro hemolysis of SEDDS was assessed based on previously described methods [20, 21]. Briefly, human whole blood was freshly diluted before the test as follows: 0.5 mL of human whole blood was added to 2 mL of sterile PBS pH 7.4 and gently shaken. Thereafter, one mL of this blood suspension was further diluted with 49 mL of PBS. SEDDS preconcentrates were diluted at different ratios (1:5, 1:10, 1:20, 1:50 and 1:100) in PBS. For the assay, fifty µL of diluted SEDDS was added to 950 µL of diluted blood in 2-mL test tubes. Therefore, the final dilution ratios of SEDDS preconcentrates in the samples were 1:100, 1:200, 1:400, 1:1000 and 1:2000. Afterwards, the test tubes were gently shaken at 300 rpm, 37 °C for 2 h (ThermoMixer C, Eppendorf, Germany), and further mixed by inversion every 15 min. The samples were then centrifuged at 825xg for 5 min at 5 °C to pellet intact erythrocytes, and the absorbance at 420 nm of supernatants containing hemoglobin released from disrupted erythrocytes were analyzed by a microplate reader (Tecan infinite M200, Austria). Triton X-100 20% served as positive control whereas PBS pH 7.4 served as negative control. The hemolysis activity as percentage was determined by Eq. 1:

1 $$\%\:hemolysis=({Abs}_{test}-{Abs}_{neg})/({Abs}_{pos}-{Abs}_{neg})$$

where Abs_test_ is absorbance of test sample, Abs_neg_ is absorbance of the negative control and Abs_pos_ is absorbance of the positive control.

### Statistical data analysis

Student’s t-test assuming unequal variances was used to assess the statistical significance of difference between two means of two samples. Ordinary one-way ANOVA with Tukey’s multiple comparisons test was used to analyze all possible pairwise means. Data are shown as mean ± standard deviation (SD), *n* ≥ 3.

## Results and discussion

### SEDDS preconcentrate preparation and characterization

Unlike conventional emulsions where a high shear stress is needed to form fine emulsions, SEDDS preconcentrate formulations often requires at least 25% surfactant [22] and the presence of a co-solvent/co-surfactant to be able to form nano- or micro-sized emulsion upon mild agitation following dilution with an aqueous phase. Three SEDDS preconcentrates were prepared using either EL35, HS15 or their combination (Table 1). Both EL35 and HS15 are approved non-ionic surfactants for use in oral and parenteral formulations. EL35 has a triglyceride-like structure with a hydrophilic head containing many ethylene glycol units (*n* = 35) and a hydrophobic tail comprised of unsaturated ricinoleate chains. HS15 consists of polyethylene glycol (*n* = 15) mono- and di-esters of the saturated 12-hydroxystearic acid and free polyethylene glycol (Table S.1). Medium-chain triglycerides like MCT have been widely used in commercial lipid emulsions for both oral and parenteral administration [23]. Medium-chain triglycerides are hydrolyzed much more quickly by lipoprotein lipase than long-chain triglycerides [24]. The mixture of MCT and MCM that served as a co-surfactant, was shown to increase the solubility of hydrophobic drugs and promote the formation of nanoemulsion upon SEDDS dilution even at a low percentage of surfactant in the SEDDS preconcentrate [25]. Cationic and anionic lipid surfactants in general are more toxic to cells then non-ionic surfactants, and their cytotoxicity profiles are dependent on their concentrations and water solubilities [26]. However, various ionic lipid surfactants like dimethyldioctadecyl ammonium bromide- an analog of DDA [27], 2,3-dioleyloxy-N-[2-(sperminecarboxamido)ethyl]-N,N-dimethyl-1-propanaminium trifluoroacetate (DOSPA), 1,2-dioleoyl-3-trimethylammonium-propane (DOTAP), 1,2-di-O-octadecenyl-3-trimethylammonium-propane (DOTMA) [28], and sodium cholate [29] have been applied in parenteral vaccine, drug and gene delivery, and proved to be safe and tolerable. In this study, low concentrations of DDA and DEO were used to prepare cationic and anionic SEDDS formulations, respectively. PG as a co-solvent was used to facilitate the self-emulsifying process of SEDDS [22, 30].

**Fig. 1:**
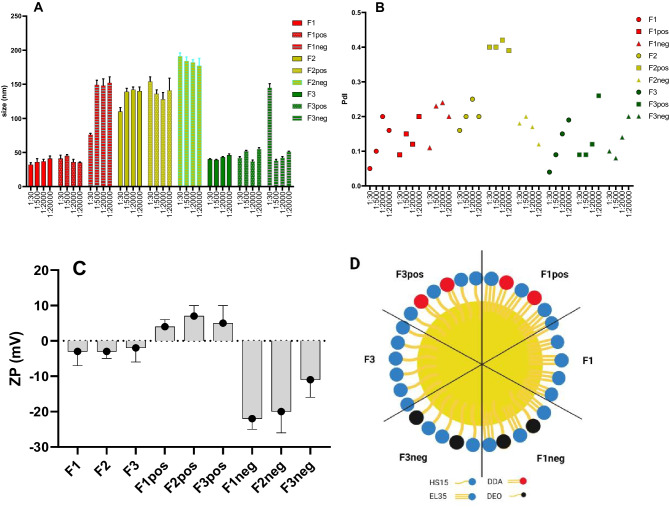
Size (**A**) and polydispersity index (PdI) (**B**) of SEDDS at different dilution ratios of 1:30, 1:500, 1:2000 and 1:20000 in PBS. For zeta potential (ZP) measurement of SEDDS diluted at 1:30 in PBS (**C**), formed emulsions were further diluted 5 times with water to reduce the high ionic concentration of PBS. Fpos = SEDDS loaded with 1% w/w didodecyldimethylammonium bromide (DDA). Fneg = SEDDS loaded with 2% deoxycholate (DEO). (**D**) Illustration of F1, F1pos, F1neg, F3, F3pos and F3neg SEDDS oily droplet surfaces. HS15 = polyethylene glycol (15)-hydroxystearate, EL35 = polyoxyl-35 castor oil, DDA = didodecyldimethylammonium bromide, and DEO = sodium deoxycholate

The F1 and F3 formulations had the same weight ratios but utilized different surfactant, EL35 in F1 and HS15 in F3 (Table 1), to evaluate the impact of surfactant type on the interaction of SEDDS and plasma proteins, and the interaction of SEDDS and cell membranes. F2 was formulated with a higher weight ratio of MCT and a lower weight ratio of a mixed surfactant (Table 1) to have larger particle size and similar surface properties as F1 and F3 to evaluate the impact of particle size on the abovementioned interactions. Upon gently mixing with PBS, F1 and F3 formed emulsions with droplet sizes 30–50 nm, whereas F2 containing 20% w/w surfactant had larger particle size of 110–150 nm. Their ZPs were in the range of -6 mV to -1 mV indicating their neutrally charged surfaces. SEDDS preconcentrates were loaded with 2% w/w DEO or 1% w/w DDA to make their surfaces negatively (ZP: -22 to -11 mV) or positively charged (ZP: 4 to 7 mV), respectively (Fig. 1C). Adding 1% w/w DDA to SEDDS preconcentrates (Fpos) did not alter the emulsion droplet size and shifted ZP to neutral-positive side. On the other hand, adding 2% w/w DEO to SEDDS preconcentrates (Fneg) resulted in significant size increases, especially in case of F1 (Fig. 1A). We supposed that DEO may intersperse between EL35 triple-tail in a similar way to the arrangement of cholesterol molecules in a phospholipid bilayer that can lead to the changes in molecular packing parameters and accordingly the curvature of the oil/water interface [31, 32]. A decrease in the curvature of the oil droplet can lead to a larger droplet radius. SEDDS were loaded with 0.1% w/w pyrene as a fluorescence marker. At this low payload, there was no significant effect on size, PdI as well as ZP.

**Table 1:** Composition of SEDDS preconcentrates

**Ingredients**	**% w/w**		
	** F1 **	** F2 **	** F3 **
MCT	30	50	30
MCM	30	20	30
HS15		10	30
EL35	30	10	
PG	10	10	10

Values are expressed in mass percentage (% w/w). MCT is a mixture of medium-chain triglycerides of caprylic and capric acids. MCM is a mixture of mono- and di-glycerides of caprylic and capric acids. HS15 is polyethylene glycol (15)-hydroxystearate and EL35 is polyoxyl-35 castor oil

We also investigated the effect of high SEDDS dilution, up to 1:20000, on emulsion droplet size. This would help to understand the possible change in size of SEDDS droplets after being diluted on SEC column. Results showed that dilution of F1, F2 and F3 (neutral and cationic) at high ratios of 1:500, 1:2000 and 1:20000 did not have significant impact on the size of emulsion droplets but there were increasing trends of PdI values (Fig. 1). There were opposite behaviors of F1neg and F3neg upon dilution where F1neg 1:500 size increased 2 times compared to F1neg 1:30, but F3neg 1:500 size greatly decreased compared to F3neg 1:30.

### Recovery of emulsion droplet–protein complexes

**Fig. 2:**
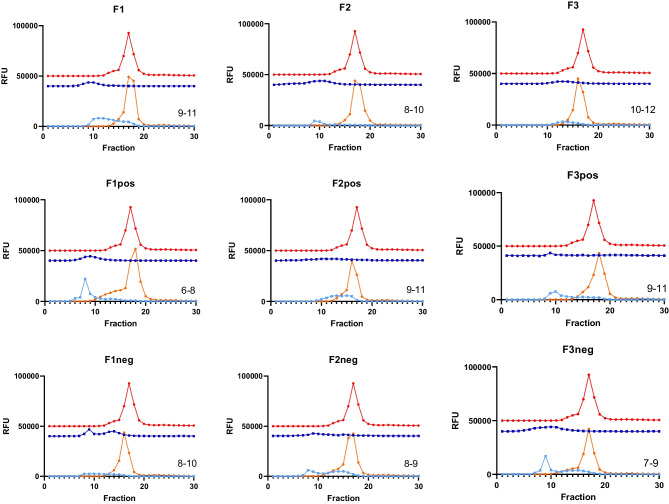
Stacked graphs of size exclusion chromatography (SEC) elution profiles of FITC labeled plasma (top and red circle), SEDDS incubated with PBS (middle and blue square) and SEDDS incubated with FITC labeled plasma (bottom: SEDDS is light blue square, and plasma is orange circle). SEDDS was detected by pyrene fluorescent signal. Plasma protein was detected by FITC fluorescent signal. The numbers indicated in each graph were the ordinal numbers of the fractions used to evaluate size, PdI, ZP and protein binding amount (µg protein/mg SEDDS) of SEDDS incubated with plasma. RFU = Relative fluorescence unit

Separation of SEDDS emulsion droplets from their mixture with human plasma was carried out on a hand-packed gravity-flow SEC column. SEDDS emulsion droplets having larger size than plasma protein passed through the SEC column faster and were eluted first as shown in Fig. 2. Gravity-flow SEC column provides a simple, robust, and low shear force approach for separation of SEDDS emulsion droplets and free plasma proteins. Alternatively, analytical ultracentrifugation (AUC) and asymmetric flow field-flow fractionation (AF4) are powerful techniques to separate SEDDS emulsion droplets from unbound proteins. However, under high centrifugal force, emulsion droplets may collide with each other and form larger droplets making it hard to determine SEDDS particle size after interaction with plasma. On the other hand, AF4 is a new technique and an excellent alternative to column-based methods like SEC and high-performance liquid chromatography (HPLC) when column chromatography cannot properly separate the analytes. It, however, is a comparatively complicated and less robust method, and its resolution is often quite poor in comparison to AUC [33].

**Fig. 3:**
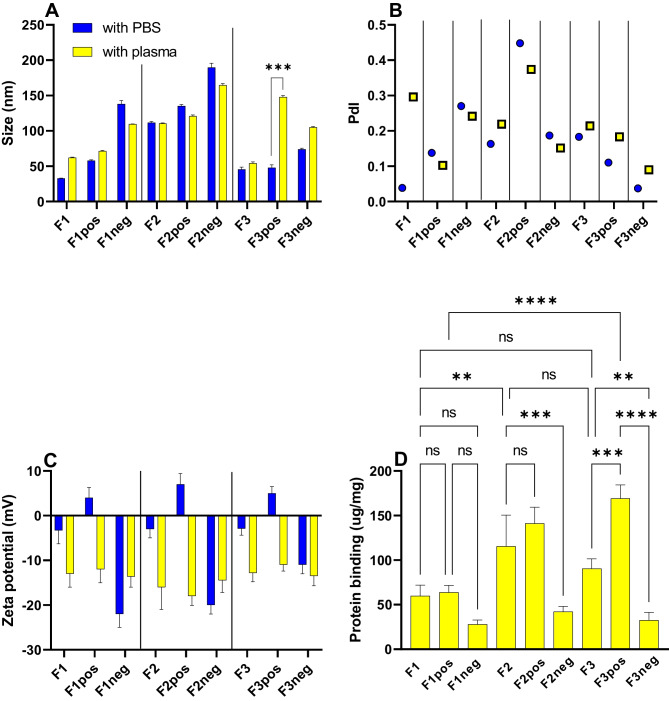
Size (**A**), polydispersity index (PdI) (**B**), zeta potential (**C**) and protein binding amount (µg protein per 1 mg of SEDDS preconcentrate) of recovered SEDDS after size exclusion chromatography (SEC) separation (**D**). PBS was used as the eluted medium. Diluted SEDDS (1:30) were incubated with either plasma or PBS (served as control) before loading onto SEC column. Blue color indicates samples incubated with PBS, while yellow color indicates samples incubated with plasma. Two to three adjacent fractions having pyrene signals and no interference with FITC labeled plasma curve were used to evaluate size, PdI, ZP and protein binding amount (mean ± SD, *n* = 2–3). Statistical significance: ns non-significant *p* > 0.1, * *p* < 0.05, ** *p* < 0.01, *** *p* < 0.001

There was a high coincidence of plasma protein SEC elution profiles detected by either FITC fluorescent measurement or BCA protein assay (Fig. S.1). Both methods showed comparable results in the quantitation and calibration ranges. FITC fluorescent measurement was used for further studies as it was sensitive and timesaving. Moreover, SEDDS and plasma separation on the column can be in situ observed and monitored using a UV lamp that is helpful in the initial stage of method development. Although an ideal separation of SEDDS and plasma protein was not achieved in some cases, fractions with high pyrene signal (recovered SEDDS) and negligible FITC signal (unbound protein) were attained in every run (Fig. 2). Those fractions were used to evaluate the particle size, PdI and ZP of recovered SEDDS and to calculate the amount of protein bound to SEDDS (Fig. 3).

Lipids can potentially interact with the BCA reagent to yield a chromophore absorbing close to 562 nm that can result in artificially high absorbance values. In SEDDS-plasma interaction experiment, SEDDS preconcentrate was diluted 1:30 in PBS followed by a further 3 times dilution upon incubation with plasma. The SEDDS-plasma mixture (50 µL) was loaded onto SEC column, eluted, and collected in fractions of 200 µL. Therefore, SEDDS preconcentrate was diluted at least 360 times in the fraction containing the highest amount of recovered SEDDS. At that dilution ratio, the interference of lipids to BCA assay would be negligible as the lipid interference created by SEDDS lipid components at 1:270 dilution ratio was just minor (Table S.2).

All SEDDS incubated with plasma had negative ZPs in the range of -18 to -12 mV regardless of their initial ZPs (Fig. 3C). This suggested the association of plasma protein onto the surface of all SEDDS droplets as confirmed by the protein binding data (Fig. 3D). Albumin (MW 66.5 kDa) is the most abundant protein in plasma accounting for ~ 55% total protein followed by globulin protein family (~ 14%, MW 93–150 kDa) and fibrinogen (4%, MW 340 kDa) [34]. The isoelectric point (pI) of albumin (4.7) is less than that of fibrinogen (5.8), β2-globulin (6.3), or ɣ-globulins (6.3–7.3) [34, 35]. Proteins are positively charged at a pH below their pI and negatively charged at a pH above their pI. For example, albumin has a net charge of -9, and fibrinogen has a net charge of -7.4 at pH 7.4 [35, 36]. That might be the reason for the higher amount of protein bound to cationic SEDDS formulations in PBS buffer (Fig. 3D). When SEDDS was loaded with 10% DDA (data not shown), they immediately formed turbid mixture upon incubation with plasma suggesting their strong interaction with plasma components. Senior et al. described similar results in their research on interaction of positively charged liposomes with blood [37]. Electrostatic attraction, however, is not the only force driving protein adsorption. Wang et al. suggested that the protein adsorption onto lipid nanoparticles was driven by multiple forces and pH dependent. Besides electrostatic attraction, van der Waals force and hydrogen bonding also play important roles in the interaction of proteins and nanoparticles [38]. Gessner et al. showed that positively charged particles (bearing basic functional groups) preferentially adsorbed proteins with pI < 5.5 like albumin, while particles with surfaces bearing acidic functional groups predominantly adsorbed proteins with pI > 5.5 [39].

Anionic SEDDS had significantly lower protein binding than corresponding neutral and cationic SEDDS (Fig. 3D). It has been reported that hydrophobic nanoparticles are opsonized more quickly than their hydrophilic counterparts, due to the enhanced absorbability of plasma proteins onto the surface of hydrophobic particles [40]. DEO structure containing one carboxylate and two hydroxyl groups is more hydrophilic than DDA, evidenced by its good solubility in water (330 g/L). The presence of DEO on SEDDS nanoemulsion droplet surfaces not only makes them negatively charged but also might render them more hydrophilic [41, 42], thus leading to lower protein adsorption. F1 formulations in both neutral, cationic, and anionic forms had lower protein binding compared to F2 and F3. The nonionic surfactant EL35 used in F1 formulations at 30% w/w seemed to induce lower protein binding to SEDDS droplet surface than HS15. F3pos stabilized by HS15 had the most significant size change and protein binding upon incubation with plasma (Fig. 3A, D). The difference in protein binding of F1 and F3 SEDDS can be derived from the higher density of polyethylene glycol (PEG) moieties in EL35 polar head structure making the surface of SEDDS stabilized by EL35 more hydrophilic. EL35 is synthesized by reacting castor oil with ethylene oxide in a molar ratio of 1:35, whereas HS15 is synthesized by reacting 12-hydroxystearic acid with ethylene oxide in a molar ratio of 1:15. Lemery et al. showed that a large volume of the surfactant polar head group limited the electrostatic interactions with proteins as a result of steric hindrance that kept the oppositely charged groups apart [26]. Woodburn et al. evaluated the plasma half-life of a hydrophobic model drug- ketochlorin photosensitizer C8KC- using EL35 and HS15 as drug delivery vehicles. The plasma half-life of the system using EL35 was 12–13 h, whereas that of the system using HS15 was 2.5 h [43]. Numerous studies have shown that a decrease in protein binding on nanoparticles will lead to a decrease in cellular uptake and an increase in the particle’s blood circulation half-life [13]. Therefore, it was possible that HS15 drug delivery systems having much shorter half-life might have higher protein binding capacity than EL35, which was in line with our observation in this study.

Particle size was shown to affect the formation of the protein corona surrounding nanoparticles in biological media. Small-sized nanoparticles can reduce protein adsorption [38, 44], but the effect can also go in the other direction where smaller-sized NPs were shown to exhibit greater protein binding [45, 46]. The impact of particle size on protein binding in this study was not clear. F2 having larger size (110 nm) than F1 and F3 (30–50 nm) showed significantly higher protein binding over F1 but not significantly different from F3. Similarly, F2pos (135 nm) showed significantly higher protein binding over F1pos (58 nm) (*p* < 0.01) but not significantly different from F3pos (48 nm), whereas F1neg (138 nm), F2neg (190 mm) and F3neg (70 nm) showed similar protein binding profiles (Fig. 3).

### Plasma membrane disruption activities of SEDDS

**Fig. 4:**
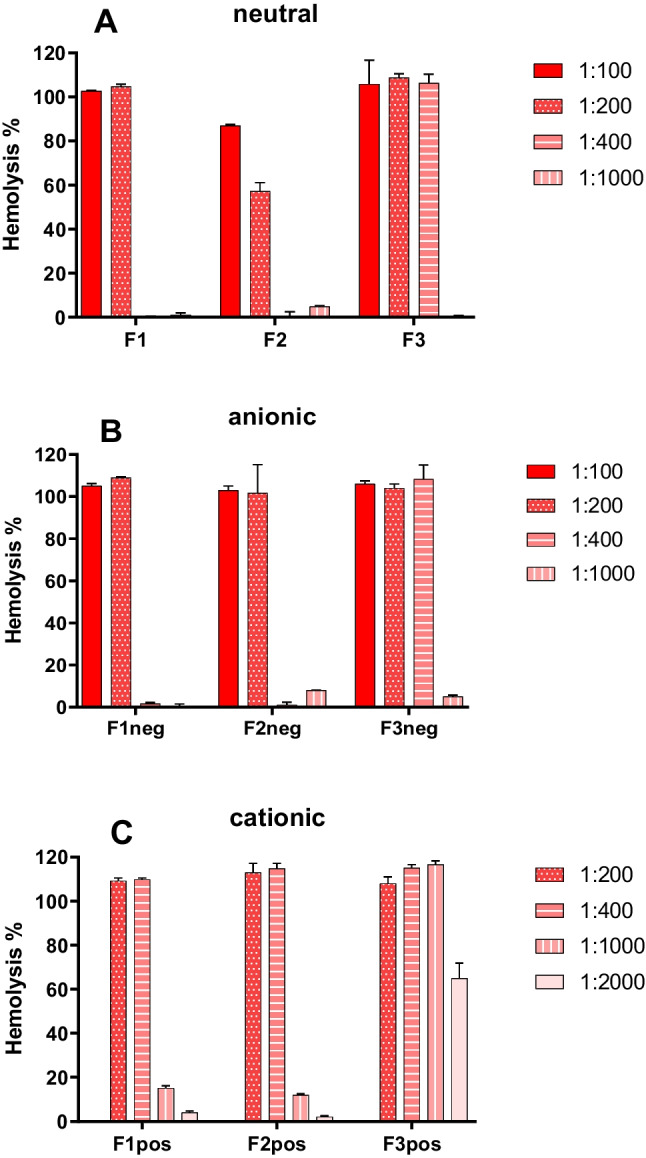
(**A**) Plasma membrane disruption (PMD) activities of 3 SEDDS formulations F1, F2, F3 at different dilution ratios 1:100, 1:200, 1:400, 1:1000 and 1:2000. Polyethylene glycol (15)-hydroxystearate HS15, used as the nonionic surfactant in F3, seemed to trigger more PMD activity than polyoxyl-35 castor oil- EL35 that is used in F1. (**B**) PMD activities of 3 SEDDS formulations containing 2% sodium deoxycholate (DEO) at different dilution ratios 1:100, 1:200, 1:400, and 1:1000. DEO seemed to increase PMD activity of SEDDS. (**C**) PMD activities of 3 SEDDS formulations containing 1% didodecyldimethylammonium bromide (DDA) at different dilution ratios 1:100, 1:200, 1:400, and 1:1000. DDA loading obviously increased the PMD activity of SEDDS compared to the unloaded or DEO loaded SEDDS

Hemolysis assay is a useful tool to evaluate the ability of SEDDS to interact and/or disrupt cell plasma membrane [20] and an indicator for endosomal escape capability of nanoparticles [47, 48]. As shown in Fig. 4, plasma membrane disruption (PMD) activity of SEDDS is concentration dependent. Cationic SEDDS clearly showed higher ability to disrupt erythrocyte membranes than corresponding neutral and anionic SEDDS formulations. Negligible hemolytic activities were observed at dilution ratio of 1:2000 for cationic SEDDS, whereas neutral and anionic SEDDS diluted at 1:400 already showed non-significant effects except for F3. HS15 having a single-tail structure and saturated fatty chain used in F3 formulation seemed to induce stronger PMD activity than EL35 having a triple-tail structure and unsaturated fatty chains used in F1. However, at 1:1000 dilution, the PMD effects of F3 and F3neg subsided (Fig. 4A, B). The PMD potency of a surfactant is related to its affinity for the membrane and the modification of the lipid membrane curvature. This is in turn related to the surfactant shape defined by the structure of its hydrophobic and hydrophilic moieties [18]. There are two main mechanisms of hemolysis by surfactants, osmotic lysis and membrane solubilization. Either mechanism, the first stage is the adsorption or binding of the surfactant onto the plasma membrane [18]. We have shown that SEDDS formulations using EL35 resulted in lower protein binding than those using HS15 (Fig. 3). This can be inferred that HS15 could interact with plasma membrane better than EL35 and thus induce stronger PMD activity. It is also evidenced that the accumulation of saturated fatty acids in plasma membrane can induce loss in membrane integrity and lead to membrane disruption [49, 50]. HS15 has lower critical micelle concentration (CMC) than EL35 (Table S.1), which might contribute to the higher PMD activity of HS15 over EL35. In general, the hemolysis activity is inversely related to CMC values, i.e. surfactants with a low CMC are more lytic [51].

This observation was in line with data from [52]. Séguy et al. showed that HS15 could induce more pronounced hemolysis activity than EL35, and EL35 and medium-chain triglycerides showed negligible hemolysis activity. In their hemolysis study, authors diluted emulsion excipients directly in whole blood whereas we diluted SEDDS in 200-times diluted blood. As such, our approach magnifies the readouts and can beneficially support the formulation development. It is worth reminding that both HS15 and EL35 are approved for use in parenteral products and have good safety profiles. HS15 has the advantage of inducing lower hypersensitivity reactions and being less immunogenic than EL35 and Tween 80- the commonly used solubilizer in parenteral formulations.

F2 stabilized by a 1:1 mixture of HS15:EL35 that was accounted for 20% w/w of the preconcentrate seemed to have the least PMD activity amongst the three formulations (Fig. 4A). The addition of DEO to F2 to make it negatively charged led to an increase in PMD activity. Negatively charged particles often showed low PMD activity [53, 54]. However, it seemed that besides the charges, the nature of the anionic moieties also decided the PMD activity. DEO at pH 7.3 has been shown to be able to increase the permeability of erythrocyte membranes to KCl that might result in colloid osmotic hemolysis [55].

Not only is a useful tool to study PMD, but in vitro hemolysis assay is also a common and important method for preliminary evaluation of cytotoxicity of parenteral products, or any blood-contacting medical device or materials. However, protocols used in the literature vary substantially and degree of hemolysis being reported as “safe” varies greatly between studies, without an actual in vivo assessment [56–58]. The use of washed erythrocytes or whole blood, and blood source can lead to dramatically different outcomes [57, 58]. While whole blood is more physiologically relevant for products intended for intravenous (IV) injection, it contains many components like lipids, proteins and electrolytes that can disguise the hemolytic activity of drugs or nanoparticles. Hemolysis induced by parenteral products in vivo is a complex event impacted by numerous factors such as drug and excipient physiochemical properties, formulation composition, concentration, administration routes (intramuscular injection, IV push, IV bolus or IV infusion), injection rate,. [56, 59]. . As a guidance, parenteral formulations with an in vitro hemolysis value lower than 10% are considered to be nonhemolytic, while those wih hemolysis values higher than 25% are considered to be at risk for hemolysis [56].

## Conclusion

We prepared three SEDDS formulations F1, F2 and F3 composed of medium-chain triglycerides as the oil phase, EL35 and HS15 as the nonionic surfactants, medium-chain mono- and diglycerides as the co-surfactant, and PG as the co-solvent. By changing the ratio of SEDDS ingredients, we can achieve neutrally charged SEDDS with desired particle size upon mild agitation following dilution with aqueous buffer. DDA (1% w/w) and DEO (2% w/w) were loaded into SEDDS preconcentrates to obtain cationic and anionic SEDDS, respectively. SEC can separate SEDDS emulsion droplets from their mixture with blood plasma. All SEDDS bound plasma protein. The degree of protein binding was determined by SEDDS surface properties, i.e., charge and surfactant type. At pH 7.4, negatively charged SEDDS adsorbed less protein than neutrally and positively charged SEDDS. SEDDS stabilized by HS15 can adsorb more plasma protein and induce more PMD activity than SEDDS stabilized by EL35. These effects were more pronounced with the HS15 + DDA combination. The addition of DDA and DEO to SEDDS increased PMD activity, and cationic DDA (1%) was more active than anionic DEO (2%). PMD activities of SEDDS were concentration-dependent and waned at appropriate SEDDS dilution ratios.

## Supplementary Information

Below is the link to the electronic supplementary material.

Supplementary Material 1

## Data Availability

The authors confirm that the data supporting the findings of this study are available within the article and its supplementary information files.

## Data availability

The datasets generated during or analyzed during the current study are available from the corresponding author on reasonable request.
