# Supplementary material for: Effects of excipients on the interactions of self-emulsifying drug delivery systems with human blood plasma and plasma membranes
Source: Drug Deliv Transl Res. 2024 Feb 27;14(11):3200–11. doi: 10.1007/s13346-024-01541-w (PMC11445307; doi:10.1007/s13346-024-01541-w)
Supplement: Supplementary file 1 — Supplementary Material 1 [file 13346_2024_1541_MOESM1_ESM.docx]

**Effects of excipients on the interactions of self-emulsifying drug delivery systems with human blood plasma and plasma membranes**

**Bao Le-Vinh^a,b^, Nguyet-Minh Nguyen Le^a,b^, Thi Nhu Quynh Phan^a^, Hung Thanh Lam^a,c^, Andreas Bernkop-Schnürch^a,*^.**

^a^ Department of Pharmaceutical Technology, Institute of Pharmacy, University of Innsbruck, Innrain 80/82, 6020 Innsbruck, Austria

^b^ Department of Industrial Pharmacy, Faculty of Pharmacy, University of Medicine and Pharmacy at Ho Chi Minh city, 700000 Ho Chi Minh city, Viet Nam

^c^ Department of Pharmaceutical Technology, Faculty of Pharmacy, Can Tho University of Medicine and Pharmacy, Can Tho city, Viet Nam

*Corresponding author:

Department of Pharmaceutical Technology,

Institute of Pharmacy, University of Innsbruck

Innrain 80/82, 6020 Innsbruck, Austria

Tel.: +43-512-507 58601 30. Fax: +43-512-507 58699

E-mail: andreas.bernkop@uibk.ac.at

# 1. Chemical structures

Table S. 1. Chemical structures and properties of SEDDS ingredients. MW = molecular weight; CMC = critical micelle concentration; HLB = hydrophilic–lipophilic balance; PEG = polyethylene glycol. HLB, MW, CMC and density values of non-ionic surfactants and oils were cited from [1,2] and product information from manufacturer. * for reference purpose; information about HLB values of cationic and anionic surfactants is rare and may vary depending on the determination method.

| **Chemical name** | **Structures** | **Properties** |
| --- | --- | --- |
| Kolliphor® HS 15 (polyethylene glycol (15)-hydroxystearate- HS15), comprised of 65%-70% of ethoxylated mono- and di-ester components, and 30%-35% of free PEGs. | 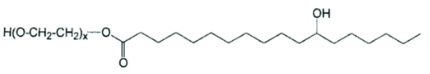  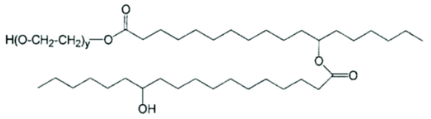 | HLB 15  MW 691  CMC 0.005-0.02%  Density 1.03 |
| Kolliphor® EL (polyoxyl-35 castor oil- EL35) | 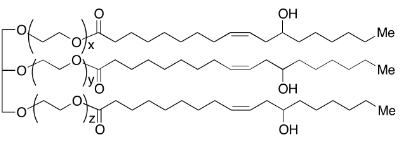  x + y +z = 35 | HLB 12-14  MW 2500  CMC 0.02%  Density 1.05-1.06 |
| Didodecyldimethylammonium bromide (DDA) |  | HLB* 18.1 [3]  MW 462.63  CMC 0.05-0.15 mM ~ 0.0023-0.0069% [4] |
| Sodium deoxycholate (DEO) |  | HLB* 16  MW 414.55  CMC 2-10 mM ~ 0.083-0.415% [5] |
| Capmul MCM (medium Chain Mono- and Diglycerides) |  | HLB 5-6  Density 0.995 [6] |
| Labrafac Lipophile WL 1349 (mixture of medium-chain triglycerides of caprylic and capric acids - MCT) |  | HLB 1  Density 0.93-0.96 |

# 2. Determine SEDDS sample dilution ratio to avoid interference in BCA assay

Lipid can potentially interact with the BCA reagent to yield a chromophore absorbing close to 562 nm that can result in artificially high absorbance values. This interference is concentration dependent, therefore, a simple way to minimize this interference is to determine the appropriate sample dilution ratio. Substances are considered compatible with the assay if the error in concentration estimation caused by the presence of the substance is ≤ 10% (Thermo Scientific’s instruction). As a precaution, we determined SEDDS sample dilution ratios to avoid any error when quantitating protein associated with SEDDS using BCA assay. Accordingly, SEDDS preconcentrates were serially diluted in PBS and diluted samples were quantitated using BCA assay following manufacturer’s instruction. Based on the absorbance signal acquired, the ‘pseudo’ protein contents of the diluted SEDDS samples were determined (Table S. 2). At dilution ratio of 1:270, the highest interference created by lipid components was 2.2 µg/mL (F1). In SEDDS-plasma interaction experiment, SEDDS preconcentrate was diluted 1:30 in PBS, then further diluted 3 times upon incubation with plasma. The SEDDS-plasma mixture (50 µL) was loaded onto size exclusion chromatography (SEC) column, eluted, and collected in fractions of 200 µL. Therefore, SEDDS preconcentrate was diluted at least 360 times in the fraction containing the highest amount of recovered SEDDS. At that dilution ratio, the interference of lipid to BCA assay is just negligible.

Table S. 2. Equivalent protein contents diluted SEDDS samples. All SEDDS preconcentrates contained pyrene.

|  | 'pseudo' protein content (µg/mL) | | | | | | | | |
| --- | --- | --- | --- | --- | --- | --- | --- | --- | --- |
| dilution factor | **F1** | **F2** | **F3** | **F1pos** | **F2pos** | **F3pos** | **F1neg** | **F2neg** | **F3neg** |
| 30 | 5.6 | 5.3 | 2.2 | 4.0 | 5.5 | 7.1 | 4.8 | 5.6 | 6.0 |
| 90 | 3.8 | 2.5 | 0.6 | 2.8 | 2.1 | 4.0 | 3.1 | 2.6 | 1.1 |
| 270 | 2.2 | 1.0 | 0.2 | 1.7 | 0.8 | -2.8 | 1.9 | 1.1 | -1.2 |
| 810 | 0.8 | 0.4 | 0.0 | 0.0 | 0.2 | -3.4 | 0.9 | 0.4 | -0.3 |

# 3. Size exclusion chromatography (SEC) elution profile of FITC labeled plasma

Figure S. 1. SEC elution profile of FITC labeled plasma detected by measuring fluorescent signal (red circles) and protein concentration (blue squares) in each fraction. Protein concentration (µg/mL) in each fraction was determined by micro-BCA assay. FITC signal pattern matched with protein concentration pattern. In fractions with low protein concentrations, BCA assay seemed to be more sensitive than FITC fluorescent measurements.

# References

[1] Y.S. Chen, Y.H. Chiu, Y.S. Li, E.Y. Lin, D.K. Hsieh, C.H. Lee, M.H. Huang, H.M. Chuang, S.Z. Lin, H.J. Harn, T.W. Chiou, Integration of PEG 400 into a self-nanoemulsifying drug delivery system improves drug loading capacity and nasal mucosa permeability and prolongs the survival of rats with malignant brain tumors, Int. J. Nanomedicine. 14 (2019) 3601. https://doi.org/10.2147/IJN.S193617.

[2] Y. Weerapol, S. Limmatvapirat, J. Nunthanid, P. Sriamornsak, Self-Nanoemulsifying Drug Delivery System of Nifedipine: Impact of Hydrophilic–Lipophilic Balance and Molecular Structure of Mixed Surfactants, AAPS PharmSciTech. 15 (2014) 456. https://doi.org/10.1208/S12249-014-0078-Y.

[3] Z.E. Proverbio, S.M. Bardavid, E.L. Arancibia, P.C. Schulz, Hydrophile–lipophile balance and solubility parameter of cationic surfactants, Colloids Surfaces A Physicochem. Eng. Asp. 214 (2003) 167–171. https://doi.org/10.1016/S0927-7757(02)00404-1.

[4] L.R. Griffin, K.L. Browning, C.L. Truscott, L.A. Clifton, J. Webster, S.M. Clarke, A comparison of didodecyldimethylammonium bromide adsorbed at mica/water and silica/water interfaces using neutron reflection, J. Colloid Interface Sci. 478 (2016) 365–373. https://doi.org/10.1016/J.JCIS.2016.06.015.

[5] U. Subuddhi, A.K. Mishra, Micellization of bile salts in aqueous medium: A fluorescence study, Colloids Surfaces B Biointerfaces. 57 (2007) 102–107. https://doi.org/10.1016/J.COLSURFB.2007.01.009.

[6] L. Vlaia, I. Olariu, G. Coneac, A.M. Muţ, C. Popoiu, S. Corina, D.F. Anghel, M.E. Maxim, S. Kalas, V. Vlaia, Development of microemulsion-loaded hydrogel formulations for topical delivery of metoprolol tartrate: Physicochemical characterization and ex vivo evaluation, Farmacia. 64 (2016) 901–913.
